# 正中开胸上腔静脉人工血管置换的程序化处理在胸部肿瘤外科治疗中的应用

**DOI:** 10.3779/j.issn.1009-3419.2017.11.05

**Published:** 2017-11-20

**Authors:** 诗杰 张, 向征 刘, 伟明 黄, 简 李

**Affiliations:** 100034 北京，北京大学第一医院胸外科 Department of Thoracic Surgery, Peking University First Hospital, Beijing 100034, China

**Keywords:** 正中开胸, 上腔静脉人工血管置换, 程序化处理, Median thoracotomy, Prosthetic reconstruction of the superior vena cava, Programmed procedure

## Abstract

**背景与目的:**

上腔静脉系统受累是局部晚期胸部肿瘤较常见的一种情况，手术可能获益，但风险极高。本研究针对正中开胸入路，提出一种程序化的手术方案，旨在优化流程，使得这一类以往认为风险极高的手术能够更加安全地实施。

**方法:**

35例胸部疾患累及上腔静脉系统，经正中开胸进行人工血管置换的患者资料，分期检查明确为局部晚期。包括肺部肿瘤16例，纵隔肿瘤19例。手术方法采用从左至右的单向推进，先游离左无名静脉起始部，阻断后切断，掀起瘤体，打开心包，用人工血管桥接左无名静脉和右心耳。游离上腔静脉近心端未受侵部分后，向尾侧牵拉肿瘤，剪开右侧纵隔胸膜，结扎切断右侧乳内血管，可以充分显露右无名静脉。向左上方牵拉瘤体，于肺门上方结扎切断奇静脉，此时可以阻断右无名静脉和上腔静脉，切除中间受侵的血管，以人工血管行右无名静脉-上腔静脉桥接，完成受侵的上腔静脉系统全部替换。

**结果:**

全组病例均顺利完成手术。术后并发症包括：心律失常6例，低氧血症5例，肌无力危象1例，心脏疝1例，真菌感染2例。2例患者死亡，死亡率5.12%，分别死于心梗和肺部感染。其余33例顺利出院。平均术后住院日15 d。在10例术前出现上腔静脉综合征的患者中，除2例术中即出现人工血管内血栓形成的患者，其余8例症状均明显改善。

**结论:**

上腔静脉人工血管置换手术经程序化的处理，规范治疗的细节，在手术操作过程中可降低手术风险，本组病例手术能够安全实施的实践也支持这一点。

## Abstract (en)

**Background and objective:**

The involvement of superior vena cava is a common condition in locally advanced thoracic tumors. Patients may benefit from the high risk operation. This study proposed a programmed procedure to optimize surgical techniques, which can facilitate the safety of operation via median thoracotomy.

**Methods:**

A total of 35 patients with thoracic disease involved superior vena cava underwent prosthetic vascular reconstruction via median thoracotomy. All patients were confirmed locally advanced without distant metastasis including 16 pulmonary neoplasm and 19 mediastinal disease. The operations proceed from left to right with one direction manner. The initial part of the left innominate vein was dissected, then cut off, so as to lift tumor, the pericardium was opened, and the left innominate vein and the right artrium were bridged with prosthetic vascular. The proximal end of the superior vena cava which not invaded was dissected and the tumor was pulled to the caudal side, the right mediastinal pleura was opened and the right inner mammary vascular was ligated and the right innominate vein was fully revealed. Stretch the tumor to left top, cut azygos vein on above the hilum, then block the right innominate vein and superior vena cava, removed involved part of blood vessels, the right innominate vein and superior vena cava was connected with prosthetic vascular. With these procedures the superior vena cava was reconstructed completely.

**Results:**

The operation was completed successfully in all cases. Postoperative complications included 6 cases with arrhythmia, 5 cases with hypoxemia, 1 case with myasthenia crisis, 1 case with cardiac hernia, and 2 cases with fungal infection. 2 patients died of myocardial infarction and lung infection respectively with a mortality rate of 5.12%. The remaining 33 cases were discharged successfully. The average postoperative hospital stay was 15 days. Of the 10 patients with superior vena cava syndrome preoperatively, 8 patients had symptoms relief except 2 cases with intraoperative intravascular thrombosis.

**Conclusion:**

We recommended the programmed procedure of prosthetic reconstruction of the superior vena cava, standardize the details of treatment, and minimize the risk during operation. The safe surgical procedures of this group of cases confirm this practice.

随着诊断水平以及人们健康意识的提高，越来越多的胸部肿瘤能够被早期发现从而得到及时救治。但仍然有相当多的患者在就诊时肿瘤的分期偏晚，这其中有很多属局部晚期，即肿瘤侵犯了相邻的重要脏器，但并未发生远处转移。上腔静脉系统受累是较常见的一种情况，可出现程度不等的上腔静脉综合征的表现。这部分患者治疗具有一定争议，单纯内科治疗效果不佳，经过评估，接受外科手术治疗，对于长期疗效及生活质量的改善都有很大帮助^[1]^。但此类手术风险极高，入路一般有侧开胸和正中开胸，而正中开胸术野显露清楚，容易控制和处理意外情况，具有一定的优势。本研究通过总结经正中开胸切除累及上腔静脉系统的胸部肿瘤的手术方法，旨在优化流程，提出一种程序化的手术方案，使得这一类以往认为风险极高的手术能够更加安全地实施。

## 1 资料与方法

### 1.1 病例资料

收集北京大学第一医院2005年1月-2015年11月因胸部疾患累及上腔静脉系统且无法单纯接受血管成型修补而需人工血管置换的患者共41例，剔除其中6例侧开胸，保留35例正中开胸患者。患者具体资料：男性28例，女性7例，年龄17岁-73岁，平均年龄（55.4±11.8）岁。出现上腔静脉综合征10例，术前均常规行胸部增强计算机断层扫描（computed tomography, CT）检查，了解上腔静脉系统受侵情况，明确无主动脉及主肺动脉受侵，即无需体外循环。血管造影有助于了解静脉内栓塞的范围以及侧支循环建立的情况，可依据病情有选择的进行。另外需行肿瘤分期检查证实无远隔转移，明确属局部晚期。由于患者依从性有差异，术前明确病理诊断的有11例，有6例进行了术前化疗或放疗。术后病理显示肺部肿瘤16例，包括肺鳞癌11例，腺癌2例，小细胞癌2例，结核1例；纵隔肿瘤16例，包括胸腺瘤5例，胸腺癌6例，淋巴造血系统肿瘤3例，炎性肌纤维母细胞瘤1例，生殖细胞肿瘤1例；其他包括肉芽肿性炎1例，肝癌转移1例，骨肉瘤转移1例。

### 1.2 手术方法

肺癌患者一般选择全麻双腔气管插管，对于纵隔肿瘤，因为瘤体大容易侵犯相邻肺组织，所以最好也采用双腔气管插管。常规正中劈开胸骨，对于胸骨受侵者需预先设计好切口能够整块切除病变骨结构和瘤体。此时可遇到3种情况，即瘤体的主体部分（或最大径）偏右、居中和偏左。对于偏左的情况，由于肿瘤只影响左无名静脉，处理相对容易，所以这里着重介绍前两种情况。

以肿瘤居中为例，由于大血管的切除重建一定涉及阻断血流，所以对于阻断时间有比较严格的要求，文献报道一般少于45 min^[2]^，我们建议不要超过30 min。当然，对于已经发生梗阻的血管，时间可比较宽松，如何尽量缩短阻断时间是减少术后并发症的关键。解剖上，左无名静脉走行长，在巨大纵隔肿瘤中最容易受侵。基于这一点，我们采用从左向右单向操作，先处理左无名静脉。向右牵拉胸腺及瘤体，尽可能游离左无名静脉起始部，阻断后切断左无名静脉。此时瘤体游离度增加，可一并切除受累的升主动脉至主动脉弓表面的心包，向上掀起瘤体后，可充分显露主动脉弓的三大分支，有利于减少喉返神经的损伤。继续打开心包，充分显露右心耳，阻断后在心耳造口，以带支撑环的人工血管（一般内径8 mm-12 mm）行左无名静脉-右心耳桥接，吻合前人工血管需肝素预处理。无论上腔静脉梗阻程度如何，侧支循环是否充分建立（开胸时还要受到一定破坏），有了这一步建立的左无名静脉回流通路，接下来可以从容地进行上腔静脉主干的阻断，而不需要过分担心阻断时间，而且也起到了降低头部静脉压的作用。

进一步于右心耳的外下方游离出上腔静脉的近心端未受侵部分。然后向尾侧牵拉肿瘤，拟游离右无名静脉（图 1）。此时，往往需要右肺塌陷，剪开右侧纵隔胸膜，结扎切断右侧乳内血管，可以充分显露右无名静脉，这一步需注意尽量保留膈神经。向左上方牵拉瘤体，于肺门上方结扎切断奇静脉，此时可以阻断右无名静脉和上腔静脉，切除中间受侵的血管，同样以带支撑环的人工血管（一般内径8 mm-10 mm）行右无名静脉-上腔静脉桥接。至此，受侵的上腔静脉系统已全部替换完成，对于纵隔肿瘤，手术接近完成，对于肺癌，还要继续行肺部手术。

**1:**
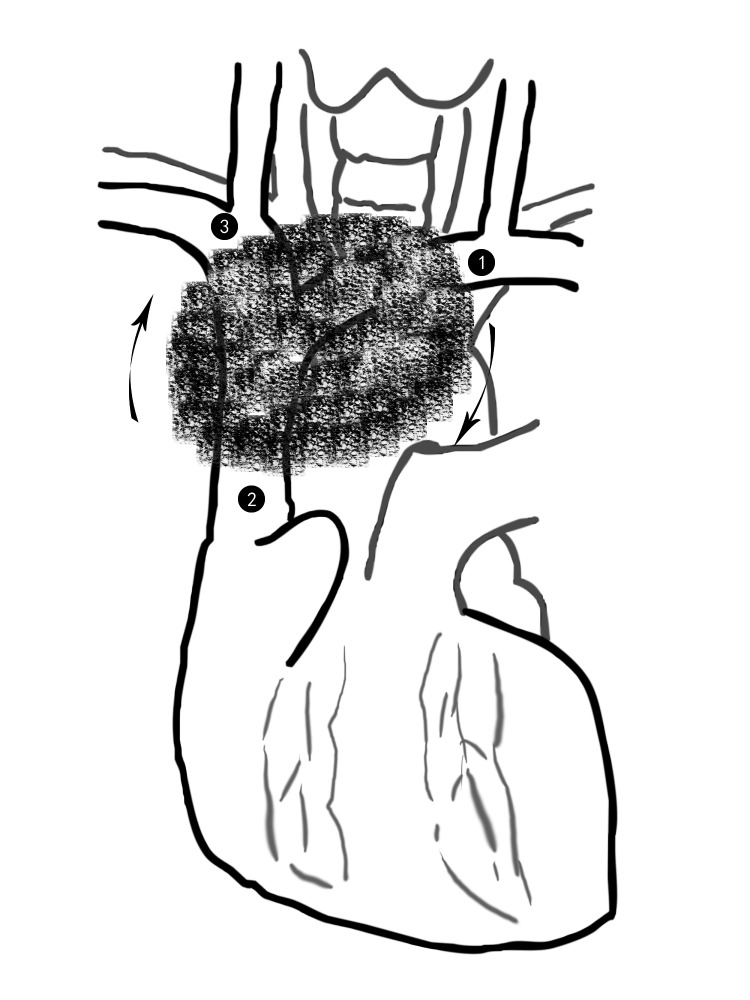
按左无名静脉→上腔静脉→右无名静脉的顺序，结合从左向右的单向推进 According to the order of the left innominate vein→ superior vena cava → right innominate vein, combine unidirectional propulsion from left to right

本组病例中，12例行单侧无名静脉置换，2例行上腔静脉主干置换，20例行双侧无名静脉分别置换，1例行左无名静脉、右颈内静脉、右锁骨下静脉分别置换。置换材料除早期有4例用自体心包外，其他均采用人工血管。具体术式包括单纯纵隔肿瘤切除9例，合并胸骨部分切除2例，合并锁骨部分切除1例，合并甲状腺部分切除1例，合并肺部分切除5例，合并肺叶切除7例，合并双肺叶切除2例，合并全肺切除8例。有2例肺腺癌患者术中即发现人工血管内血栓形成，拆除吻合线，清除血栓后重新吻合。对于膈神经受侵需切除者，应做膈肌折叠术。若心包缺损大，可行补片或织网修补。

## 2 结果

全组病例均顺利完成手术。手术时间299 min-1, 240 min，平均（586.7±193.9）min；手术出血200 mL-7, 000 mL，平均1, 882 mL。术后并发症包括：心律失常6例，低氧血症5例，肌无力危象1例，心脏疝1例，真菌感染2例。2例患者死亡，死亡率5.12%，包括1例双侧人工血管置换合并右上肺叶切除，病理为鳞癌，于术后第1天死于心梗；另1例双侧人工血管置换合并右全肺切除，病理为鳞癌，于术后第8天死于肺部感染。其余33例顺利出院。平均术后住院日15 d。在10例术前出现上腔静脉综合征的患者中，除2例术中即出现人工血管内血栓形成，其余8例症状均明显改善。

## 3 讨论

对于侵犯上腔静脉的胸部恶性肿瘤患者，单纯内科治疗、内科联合放射治疗、单纯上腔静脉病变切除术、旁路分流术^[3, 4]^、血管内皮支架术等治疗，都只能暂时缓解上腔静脉梗阻症状，达不到根治的目的，预后较差。因此在解除上腔静脉梗阻症状的同时，积极处理原发病灶，是治疗关键。随着血管外科技术在胸外科的应用，有很多的外科医师尝试上腔静脉人工血管置换，并取得良好的效果。尽管如此，面临累及上腔静脉系统的病变，由于手术风险极高，死亡率4.5%-14%^[1, 5, 6]^，远期效果不理想，大多数胸外科医师还是会望而却步，或者感到无从下手。

本文介绍了替换上腔静脉系统的程序化步骤，其特点是从左向右单向操作，依次处理左无名静脉起始部、上腔静脉近心端、右无名静脉起始部，奇静脉通常需要切断，膈神经根据是否受累决定能否保留。要充分利用右心耳，最终以双侧人工血管分别汇入右心房。事实上，很多文献都提到了首先进行静脉转流以降低静脉压力，多以人工血管桥接左无名静脉和右心耳，但后续的操作均无规律性的描述，这是本文要重点强调的。需要注意的是以下几点：①人工血管的选择，口径过大，术后血流缓慢，增加血栓风险；口径过小，不足以改善梗阻，所以我们多选用内径8 mm-12 mm带支撑环的人工血管^[5, 7, 8]^。对于术后是否应用抗凝治疗一直存在争议，有作者认为抗凝治疗并非必需^[9]^。我们的经验还是术后根据引流量及颜色尽早开始抗凝治疗，并由注射低分子肝素抗凝过渡为口服华法林钠片治疗^[10, 11]^。②无名静脉起始部的游离，若肿瘤侵犯较广泛，可能需要增加颈部切口，甚至切断锁骨来显露锁骨下静脉和颈内静脉，这种情况右侧发生的可能性更大。③上腔静脉近心端的阻断，需注意避免损伤窦房结。④有些患者高凝状态，人工血管在术中就可能发生血块阻塞，需警惕。⑤术中可依据血管受侵情况决定哪根无名静脉需要置换，有时为减少手术时间和风险，对于双侧梗阻时也可只置换一侧，对于完全梗阻的血管也可考虑直接封闭而不用置换，但原则上还是尽量恢复双侧通路，减少术后上肢头面部水肿的程度或其他由于静脉压力过高引起更严重的并发症^[12]^。

本组病例中2例为转移瘤，1例为肝癌术后3年，1例为右股骨骨肉瘤术后4年。2例均无原位复发，且除纵隔转移外未见其他部位转移，化疗无明显效果，1例已经出现颜面部水肿。经多学科讨论认为患者有可能通过手术获益，再评估其心肺功能后才决定手术治疗。近来也有对于转移瘤累及上腔静脉进行人工血管置换，患者获益的报道^[13]^。

本文只是总结了此类手术的经过优化并程序化的手术步骤，对于有手术指征的患者可以减少术中与外科操作相关的风险，但对于上腔静脉受累的病变，由于分期偏晚，患者全身状况往往不佳，手术风险要远远大于常规胸外科手术，这些客观存在的事实是需要重视的。

对于累及上腔静脉系统的胸部肿瘤，外科治疗是有意义的。但手术风险极高，面对病变往往无从下手，正是基于此，我们第一次提出血管置换手术程序化的处理，规范治疗的细节，可以在手术操作过程中降低风险，本组病例手术能够安全实施的实践也支持了这一点。随着研究的深入，可能会使更多的局部晚期胸部肿瘤患者通过外科治疗获益。
